# Medical students in Russia evaluate the training during the COVID-19 pandemic: a student survey

**DOI:** 10.1186/s12909-021-02997-x

**Published:** 2021-11-02

**Authors:** Laysan Mukharyamova, Arina Ziganshina, Aleksandr Zhidjaevskij, Liana Galimova, Maksim Kuznetsov

**Affiliations:** 1 Head of Department of History, Philosophy, and Social Science, First Vice-Rector, Kazan State Medical University, Kazan, Russia; 2 Department of Pediatrics, Kazan State Medical University, 420012 Butlerova St. 49, Kazan, Tatarstan Russia; 3 Department of Internal Medicine, Kazan State Medical University, Kazan, Russia; 4 Faculty of General Medicine, Kazan State Medical University, Kazan, Russia; 5 Department of Epidemiology and Evidence Based Medicine, Kazan State Medical University, Kazan, Russia

**Keywords:** SARS-CoV-2, Medical education, Evaluation, Online training, E-learning

## Abstract

**Background:**

The aim of the study was to obtain feedback from medical students in Russia regarding their e-learning experience during COVID-19 Pandemic.

**Methods:**

Thirteen thousand forty students from 46 Medical Schools in Russia completed an original evaluation form validated by 6 experts. Criterion and construct validity were determined in a pilot study (*n* = 46). The study design was based on the use of Google Forms. Participants used the Visual Analog Scale from 1 to 10 to assess the level of knowledge acquired.

**Results:**

95.31% of medical schools in Russia switched to e-learning during the Pandemic. 39.8% of the students stated that the time to prepare for the class has doubled. For 19.9% of them, it increased by one third, while 26.6% did not report any changes. 38,4% of the participants are satisfied with particular elements of e-learning, 27.5% like such a format, 22.9% do not like it, and 11.2% could not answer the question. The average scores for the knowledge assessment were 5.9 for the humanities, 6.1 for fundamental science, and 6.0 for clinical training.

**Conclusions:**

The most important findings are increased self-instruction time, insufficient knowledge gained and territorial and socio-economic inequalities within the country. Meanwhile, most students favor distance learning or its particular elements. Consequently, medical education leaders in Russia should consider the implementation of blended training in medicine taking into account specific regional factors, ensuring its effectiveness at all stages.

## Background

With more than 231 million people worldwide infected with COVID-19 and more than 4 million deaths since the pandemic began [1], many governments have implemented several public health policies, such as “social distancing” and “lockdowns” of varying severity for entire populations [2]. Accordingly, the COVID-19 lockdown could engender disruption to lifestyle behaviors, thus impairing mental wellbeing in the general population [3, 4].

The pandemic has presented enormous challenges to national education systems. Institutions worldwide were forced to discontinue face-to-face training and required to urgently modify curricula for online learning. In addition, the lives of students were disrupted in numerous ways [5], including considerable impacts on their wellbeing. Without exception, medical schools around the world were forced to adapt the curriculum to new realities while ensuring the highest possible quality of education. With regard to the Russian Federation, universities were firstly recommended to pursue all student-teacher interactions exclusively using electronic information and educational technologies [6]. Later, the Ministry of Health issued instructions on rescheduling the practical training of students to provide them with the opportunity to participate in activities aimed at preventing and reducing the spread of infection [7]. Finally, in May 2020, according to the mutual order of the Ministry of Science and Higher Education and the Ministry of Health, the heads of educational institutions have been instructed to send senior students specializing in general medicine, pediatrics, preventive medicine, dentistry and nursing to practical training in hospitals [8].

Curriculum reforms promoting active learning, undertaken by numerous educational organizations around the world over the past decade, have facilitated an easier transition to an online mode of training [9, 10]. The ‘flipped classroom’ and other hybrid teaching methods suitable for an online format have enabled the transformation of small group hands-on sessions into videoconferences [11]. The so-called “virtual classes” are now being held with the use of Internet resources for video conferencing (Google Hangouts Meet, Zoom, Slack, CiscoWebEx), course management systems (Elias, Moodle), and by means of platforms for unified communication and collaboration, such as Microsoft Teams, Google Classroom, Canvas, and Blackboard [12]. In Russia, the transition to online learning was facilitated by the fact that, according to state educational standards, each organization had to develop an electronic information and educational environment since the 2010s.

Despite this, Russia remains skeptical about distance learning, and fears regarding the persistence of such a format of education are being expressed [13]. Medical schools were faced with the urgent issue of organizing remote training, and ensuring proper clinical skills development in future physicians. This was particularly challenging for education delivery in disciplines that previously had always been solely offline. Based on the fact that students have the potential to influence the course of the educational process and shape their learning, it is of great importance to obtain their feedback on the online learning experience. The present study was conducted with the aim of investigating the perspectives and effectiveness of remote training programs for medical undergraduate trainees in Russia, in order to facilitate the improvement of the format and to assess its feasibility for future use.

## Methods

### Participants and data collection

In the course of a cross-sectional study 13,040 medical students from 46 institutions of Russia have been surveyed. The main objective of the research was to investigate the satisfaction of undergraduates with the effectiveness, relevance, and benefits of training during the pandemic. Therefore, the questionnaire was designed with this objective in mind. Among those who have participated in the study, 65.9% were 1st – 3rd year students, while 34.2% were 4th – 6th year students. 77% were women, and 23% were men. The student survey was initiated by the Center for Research in Medical Education and the Student Council on Quality of Education of Kazan State Medical University. The study design was based on the use of Internet survey technology through Google Forms. The completion of the questionnaire was strictly anonymous. The information about the survey was communicated to medical undergraduates through social networks. In our study, this approach allowed to minimize the influence of university administrations on student responses.

The information obtained from Google forms was quantitatively analyzed. Later, the data was exported to Excel sheets. The absolute and relative frequencies of various demographic variables and responses from the students were tabulated. Statistical analysis and visualization of obtained data were performed using R 4.1.0 (R Foundation for Statistical Computing, Vienna, Austria). To test the hypothesis of independence between two categorical variables chi-square test was used. The Mann-Whitney test with Monte Carlo approximation of test statistic distribution (10,000 replicates) was used to test the difference of ordered variable distribution locations between the groups (coin 1.4.1 package). For overrepresentation analysis, the weighted logarithmic odds were estimated as described by Monroe et al. (2008) [14].

### Study questionnaire

We developed a survey sheet in Russian containing 27 questions. The questionnaire was content validated by 6 experts. Criterion and construct validity were determined in a pilot survey of students (*n* = 46) who belonged to different years of study and medical specialties. The Visual Analog Scale (VAS) of 1 to 10 was used to assess the level of knowledge acquired by the participants.

### Ethical approval

Approval was issued by the Institutional Ethics Committee of Kazan State Medical University (Permit #2 from February, 16, 2021). Informed Consent to participate in the study was obtained electronically from all respondents at the beginning of the survey. To increase participation, students were notified that the results would only be used in a generalized form and that data from particular universities would not be published. The information collected was kept confidential and no identification was used during the analysis.

## Results

According to the analysis of the results of the survey, the vast majority of medical universities shifted to a distance learning (DL) format immediately or several days after the corresponding order, as stated by 95.31% of the participants. Very few students (2.75%) reported that only lectures became remote, while practical classes were held in the usual mode. For 1.82% of the undergraduates, the mode of training did not change. To determine the readiness of students for DL, we asked if this educational format was new to them. Most of the undergraduates (79.7%) responded that they had never studied remotely before. Some participants (12.3%) noted that their universities frequently used distance technologies for teaching and assessment. It is important to note that the majority of the students (83.4%) began studying on an educational portal of their school, while almost half of those surveyed (48.5%) said they used messengers and video conferencing, and only 8.7% began using mass open online courses (MOOCs).

Students were using a variety of devices for online learning. Overall, in the sample, most of them were attending virtual classes and doing assignments on a laptop (41.0%). With minimal difference, a smartphone took the second place among the preferred tools for DL (35.9%), followed by a personal computer (PC) (17.4%) and a tablet (5.7%). The geographical distribution for the federal districts of Russia is presented in Table 1.

**Table 1:** Most commonly used devices for online learning in different federal districts of Russia, 13,040 respondents, 2020

Federal district	Mobile phone, number, and % of students	Laptop, number, and % of students	PC, number, and % of students	Tablet, number, and % of students
Central	194/892 (21.8%)	436/892 (49.0%)	193/892 (21.7%)	69/892 (7.5%)
Far Eastern	485/1644 (29.5%)	801/1644 (48.7%)	313/1644 (19.0%)	45/1644 (2.7%)
North-Western	79/355 (21.9%)	198/355 (55.0%)	54/355 (15.0%)	24/355 (8.1%)
Volga	358/1285 (27.8%)	607/1285 (47.1%)	241/1285 (18.9%)	79/1285 (6.2%)
North Caucasian	557/819 (68.0%)	149/819 (18.2%)	64/819 (7.8%)	49/819 (6.0%)
Siberian	364/1886 (19.3%)	1007/1886 (53.4%)	389/1886 (20.6%)	126/1886 (6.7%)
Ural	200/777 (25.7%)	369/777 (47.5%)	182/777 (23.4%)	26/777 (3.3%)
Southern	1841/3638 (50.6%)	1037/3638 (28.5%)	506/3638 (13.9%)	254/3638 (7.0%)

**Fig. 1:**
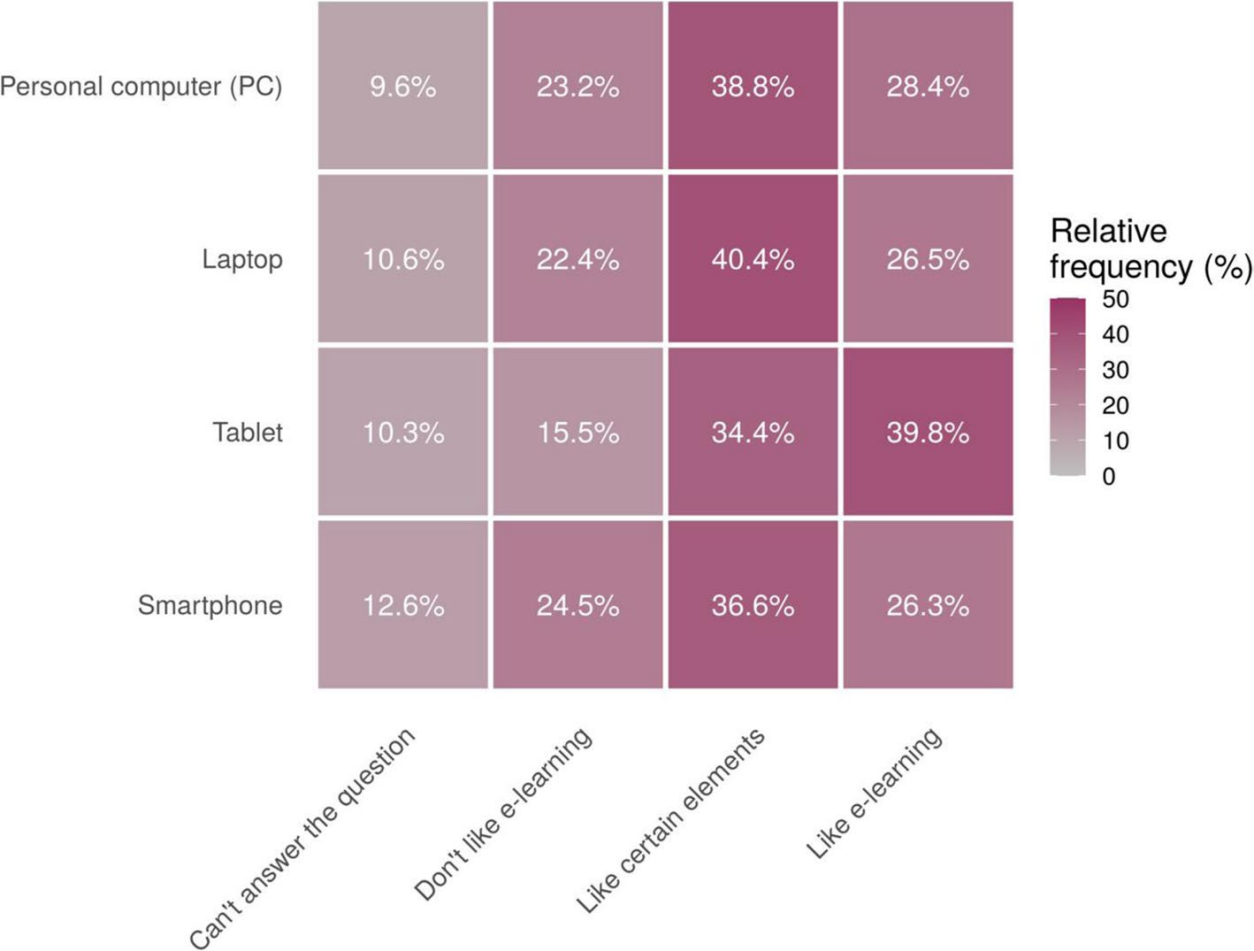
Relative frequencies of overall student satisfaction with DL depending on a device used to attend classes

When answering the question “Do you like learning through distance?” 38,4% of the surveyed stated that they are happy with certain elements of remote training, 27.5% - like such a format, 22.9% - do not like it, and 11.2% - found the question difficult to answer. Fig. 1 shows the relative frequencies of overall student satisfaction with DL depending on a device used to attend classes (*p* < 0.0001).

**Fig. 2:**
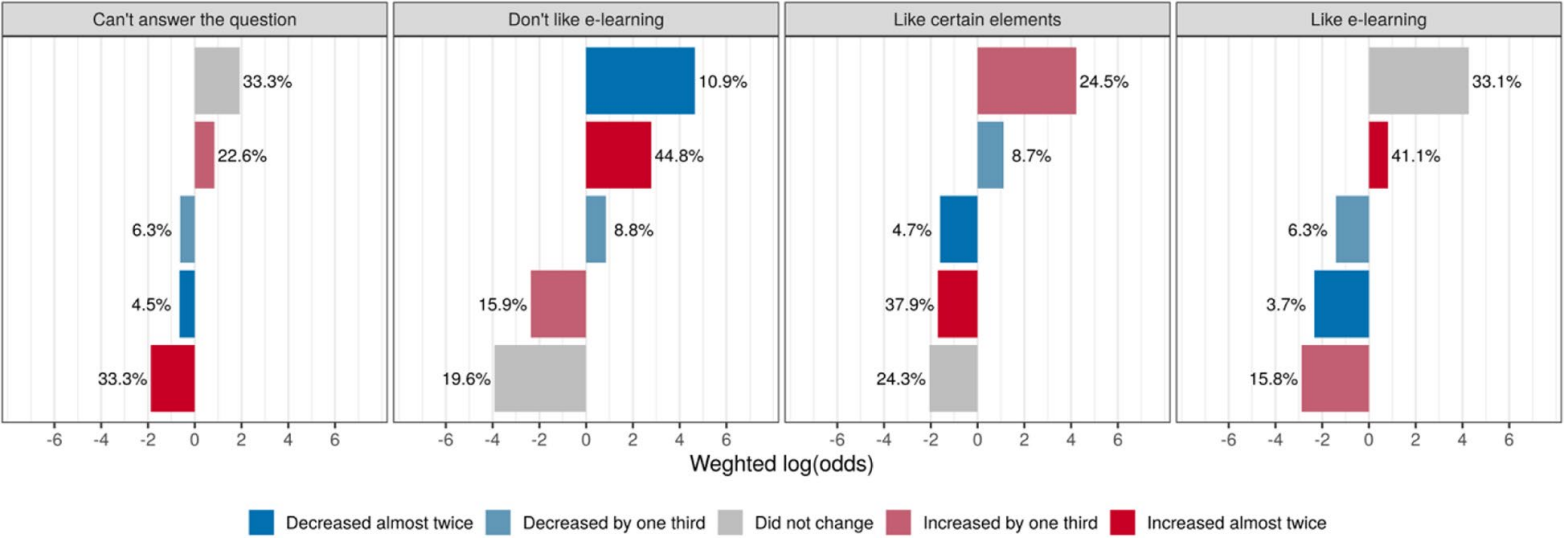
The distribution of odds if respondents who noticed a change in the duration of preparatory work favor or dislike DL

When asked about how the time to prepare for classes has changed after the transition to DL, most of the students noted that the time for independent studies has doubled (39.8%) or increased by one-third (19.9%). Meanwhile, over a quarter of undergraduates who did not notice significant changes (26.6%). It is important to note that, on this basis, the time spent for preparation for the classes decreased by half for 5.8% and by one third for 7.8% of the respondents. An overrepresentation analysis using weighted logarithmic odds was performed to understand whether the increase in independent study hours was a positive or negative finding. Fig. 2 shows the distribution of odds for whether respondents who noticed the change in the duration of preparatory work favored or disliked DL.

At the time of the coronavirus outbreak, most of the surveyed students (88.6%) were not attending clinics for educational purposes. Only undergraduate members of the *Medical Volunteers* All-Russian Public Organization continued to perform their activities in health institutions (5.8%). Meanwhile, there were trainees who responded that they had live classes (5.7%) or practical training (1.1%) in clinics during the pandemic.

We asked students to evaluate the level of knowledge they received during DL in the humanities, fundamental, and clinical (professional) disciplines. The average scores were 5.9 for socio-humanitarian subjects, 6.1 for fundamental science and 6.0 for clinical training out of a maximum of 10.

**Fig. 3:**
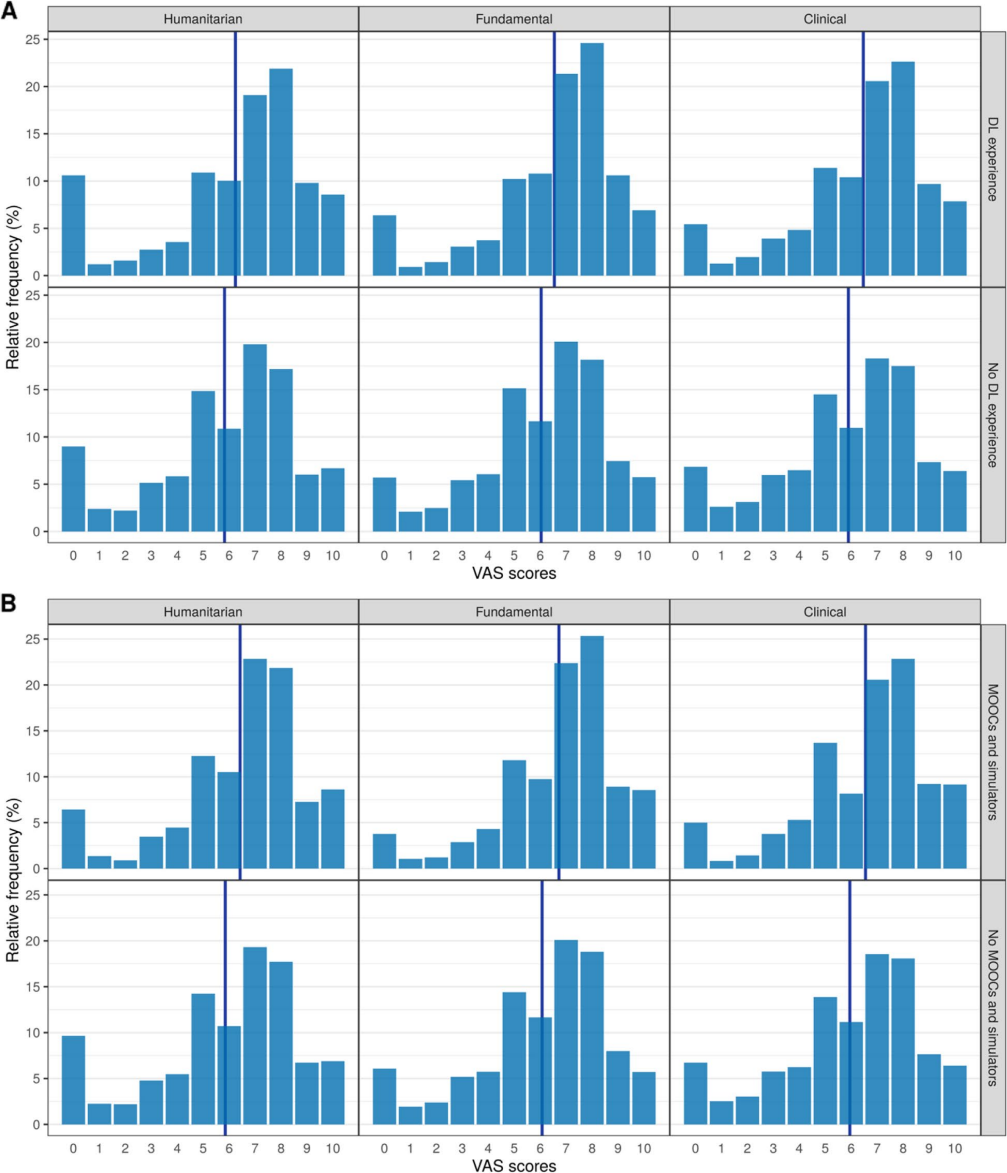
Relative frequencies and average values (marked as blue lines) for VAS scores conditioning in groups of students: A – depending on the DL experience, B – depending on using MOOCs and simulators (*p* < 0.0001).

The VAS scores for different categories of disciplines were analyzed to investigate possible associations with the lack of online learning experience and with the use of simulators and MOOCs by the Universities. The relative frequencies and average values for the conditioning of VAS scores in the groups of students are presented in Fig. 3 (in all cases the differences are statistically significant, *p* < 0.0001).

Regarding the evaluation of the educational experience gained in the new reality, 33.3% of the students believe that lectures can be transferred to a DL format. Others suggest that remote training can be implemented for entire disciplines (21.0%). Meanwhile, 8.4% of students are confident that such practice should be maintained unconditionally for the implementation of the entire curriculum. According to the respondents, the main advantages of distance technologies are the ability to learn at one’s own pace (62%), saving time and money on travel (60%), as well as developing self-control skills (54.4%). There were opponents of online learning, who noted that it reduces the effectiveness of training (10.1%). A significant proportion of undergraduates responded that it is impossible to master medicine remotely without actual presence in clinics (20.2%). Some students believe that they are not ready for distance education due to the fact that it is difficult for them to study independently without constant supervision from a teacher (3.9%). A smaller percentage of the students have not yet developed a solid opinion on this issue (3.1%) (Table 2).

**Table 2:** Student opinions on the best use of online learning, 13,040 respondents, Russia, 2020

Opinion	% of students
Lectures should be in an online format	33,3
Remote training can be implemented for entire disciplines	21,0
Online learning should be maintained unconditionally	8,4
Online learning cannot be maintained	10,1
Training in clinics should be a priority	20,2
I am not ready to study without the direct supervision of a teacher	3,9
No solid opinion	3,1

## Discussion

The present study reports the final results from 13,040 participants who responded to our survey, which showed that the overwhelming majority of medical students in Russia were trained with the predominant use of online learning methods during the COVID-19 pandemic. Therefore, understanding the thoughts of medical students about remote training would help improve the curricula in Russian universities.

A major finding in this study was the relatively poor evaluation score for the level of knowledge gained, regardless of the field of study: clinical, natural sciences, or social humanities. Our assumption that it is more important for medical students to acquire clinical skills at the bedside, while other sciences can be learned remotely, was not confirmed. A previous study carried out in India revealed that more than half of the students surveyed reported that they could not get more benefit from online classes compared to traditional learning. In addition, more than 50% of the participants confirmed that the level of discipline was affected due to continuous online learning [15]. The present results are in accordance with those of another previous survey, where only 8.8% of the students of Pakistani medical schools agreed that the classes offered during the pandemic were quite effective [16].

To better understand the reasons for such surprising results, associations between VAS scores with the presence of an online learning experience and those with the use of simulators and MOOCs by schools were investigated. Our hypothesis that the level of knowledge would be affected by the lack of experience of studying remotely was confirmed by the results of statistical analysis for all 3 categories of disciplines, suggesting that the importance of proper training on how to study online is not overstated.

The survey showed that medical schools in Russia did not make sufficient use of MOOCs during the pandemic, despite the fact that there are such resources available on the platforms of the World Health Organization, Coursera, Harvard Medical School, London School of Hygiene and Tropical Medicine, University of Pittsburgh, as well as on Russian Open Education platforms and on the educational portal of Sechenov University. Students did not indicate whether they had access to paid resources that present not only the most interesting lectures and videos, but also interactive simulators. The experience of Kazan Medical University in the use of the Cyberpatient platform showed that student satisfaction and motivation increase with the use of virtual simulators [17]. The results of the correlation analysis within the present study are concordant with findings obtained previously, confirming our assumption that the insufficient use of MOOCs and simulators has contributed to the poor VAS scores.

During the pandemic, some of these platforms, such as the online simulation platform called CyberPatient, were open to medical students in all countries for free. The use of such modern gaming technologies is important for medical education, not only during a pandemic, but also within conventional education. However, their high cost creates barriers to access for universities. It seems that the acquisition of the rights to use such resources should become part of national strategies for the advancement of medical education.

It should be noted that the geographical distribution of the results by regions of Russia illustrates the existing heterogeneity in terms of the availability of essential facilities and equipment to study online. In the Southern and North Caucasus federal districts, slightly more or less than half of the students did not find it possible to use a PC or laptop for online learning. The current findings might indicate two circumstances. Firstly, the case may be that not all undergraduates are equipped with multiple devices for studying, with most only having smartphones. Secondly, that there is insufficient availability of the Internet, other than via mobile connectivity in particular regions of Russia, which can be explained by the different speeds of digital infrastructure development in different parts of the country. A similar survey conducted in India showed the opposite proportion for the country, with more than 70% of trainees prone to attending classes with the use of mobile devices [18].

The relationship between the use of smartphones to attend classes and satisfaction with remote learning has been hypothesized to be negative. However, statistical analysis has not confirmed this statement. In contrast, almost two-thirds of the respondents who studied using a smartphone favor at least some elements of DL. Overall, while differences in student satisfaction with DL depending on their use of different devices were seen to be statistically significant, they cannot be considered practically meaningful due to minimal variance in percentages.

We also found that the new learning conditions have led to variations in academic workload. According to almost two-thirds of the respondents, the transition to remote training has led to a considerable increase in the time to self-study. Statistical analysis showed the growth of the workload for more than 60% of those students who dislike DL, suggesting that the finding should be added to the list of disadvantages of online education delivery.

According to the data obtained, there are certain benefits of DL from the student perspective. These include more personalized training, less time and money spent on travel, and the promotion of self-control in the course of studies. It is important to note here that slightly less than one-third of the respondents fully enjoy the new way of studying fully and advocate for adherence to the ‘new normal’ even beyond the pandemic. Almost the same number of trainees support certain elements of the modified training. In particular, one-third of them prefer online lectures to live ones. Meanwhile, another third of the participants are against online learning or could not shape their opinion on the matter.

Addressing the international experience, the overwhelming majority of Indian undergraduates prefer the traditional (50%) or blended (39,5%) mode of training [15]. Therefore, Indian medical students who favor DL are in the minority [18]. At the same time, trainees in Pakistan have developed a solid opinion against studying online as stated by 86,4% of the study participants [16].

Feedback obtained from learners appears to be one of the most useful tools for the refinement of existing teaching strategies, making them more efficient from the perspective of students [15]. Universities and teachers in Russia should consider the findings of the present study when developing educational programs, focusing their efforts on creating quality content on electronic educational platforms and developing assessment tools that require students to be active and creative.

Effective vaccines against SARS-CoV-2 infection, which are commonly considered as one of the most successful ways to prevent and control the disease [19], are currently available for all adults in Russia. However, vaccine hesitancy, especially in younger age groups, remains a significant barrier to overcome in achieving effective herd immunity [20]. Therefore, national programs of health education should target student and teacher populations aiming to resume pre-Covid levels of normality relating to live clinical training in a hospital setting.

### Limitations of study

A few areas of concern were not described in the study, including the level of stress students have experienced due to the pandemic, the quality of preliminary training, the availability of technical support, mentoring provided by schools, the speed of the Internet, and related issues. In addition, the online-advertised study could have resulted in volunteer bias.

## Conclusions

The present study was an effective tool for gathering authentic feedback on the suddenly changed mode of training from medical students from all over the country. It reflects on previously unrevealed elements of learning during the pandemic, thus allowing educators to overthink the feasibility of the newly introduced components for future times. The most important findings include increased self-instruction time, insufficient knowledge gained as well as territorial and socio-economic inequalities within the country. Meanwhile, most students favor DL or some specific elements of DL, such as online lectures, and vote for maintaining remote training even beyond the pandemic, explaining this by the fact that distance technologies allow individualized learning, savings of time and money, as well as helping to foster self-control.

Consequently, considering the growing popularity and enhancement of online learning, leaders in medical education in Russia should contemplate the implementation of blended training in medicine. This should consider specific regional factors while also ensuring the effectiveness of its components at all stages.

Moreover, it is important to provide proper instruction, ongoing feedback and communication with faculties when introducing any changes to the training routine.

## Data Availability

The data supporting the findings of this study is available from the corresponding author, AZ, upon reasonable request.

## Abbreviations

COVID-19: Coronavirus disease 2019
DL: Distance learning
MOOCs: Mass open online courses
PC: Personal computer
VAS: Visual Analog Scale
